# Chairside Restorations of Maxillary Anterior Teeth with CAD/CAM Porcelain Laminate Veneers Produced by Digital Workflow: A Case Report with a Step to Facilitate Restoration Design

**DOI:** 10.1155/2019/6731905

**Published:** 2019-04-04

**Authors:** Elif Öztürk Bayazıt, Murat Karabıyık

**Affiliations:** ^1^Hacettepe University, Faculty of Dentistry, Department of Restorative Dentistry, 06100 Sıhhiye, Ankara, Turkey; ^2^32 Diş Dünyası Ağız ve Diş Sağlığı Polikliniği, 34940 Tuzla, İstanbul, Turkey

## Abstract

Since the introduction of porcelain laminate veneers in the early 1980s, the anterior esthetic rehabilitation with PLVs has been provided with a conventional restorative technique for many years. Recently, a new concept named digital workflow has been raised that will lead to the abandonment of conventional procedures during the fabrication of PLVs. Digital workflow is a new concept facilitating applications by adapting digital systems to clinical applications, including intra- and extraoral photographs, diagnostic wax-up, mock-up, intraoral camera, and use of the CAD/CAM device. The aim of this case report is to describe the clinical procedures of porcelain laminate veneers using digital workflow with a facilitating step in a single session and to evaluate the PLVs after one year of clinical service.

## 1. Introduction

During the last few decades, the demand for the computer-aided design and manufacturing systems in dentistry has dramatically increased because of the rapid developments in computer technology and science [1]. The idea of CAD/CAM was emerged with the aim of creating excellent restorations with the least possible error by the computer, as opposed to the conventional manufacturing techniques by free hand, which is prone to numerous subjective failures [2]. CAD/CAM systems can be categorized as either chairside or laboratory systems [3]. Chairside CAD/CAM systems allow the clinicians in private offices to independently design and also machine dental ceramic restorations within a few hours during a single visit [1, 4]. Inlay, onlay, porcelain laminate veneer, crown, and bridge can be produced with today's CAD/CAM systems.

The clinical technique for porcelain laminate veneers includes bonding very thin restorations to tooth adhesively in order to correct an unesthetic appearance of the anterior teeth [5]. The main reason why PLVs are so demanded restorations is that they are very minimally invasive restorations and very good esthetic results can be achieved with them [6]. With the improvements in CAD/CAM systems, such thin restorations with a thickness of 0.3 - 0.7 mm could be created almost perfectly with the help of computer design [7, 8]. Excellent esthetic results can be provided by these restorations especially with the use of digital workflow in recent years [9].

Several medium-to-long-term clinical studies have evaluated the clinical performance of PLVs produced by conventional techniques. The success of conventional PLVs in these clinical trials ranges from 100% to 67% [6, 10]. However, to the authors' knowledge, only limited clinical data about the porcelain laminate veneers (PLVs) produced by CEREC CAD/CAM chairside systems have been reported and no long-term clinical results have existed. Therefore, the aim of this case report is to explain the clinical procedures for fabricating PLVs with CAD/CAM in a single session using digital workflow with a facilitating step and to report a year of clinical results.

## 2. Case Report

**Figure 1:**
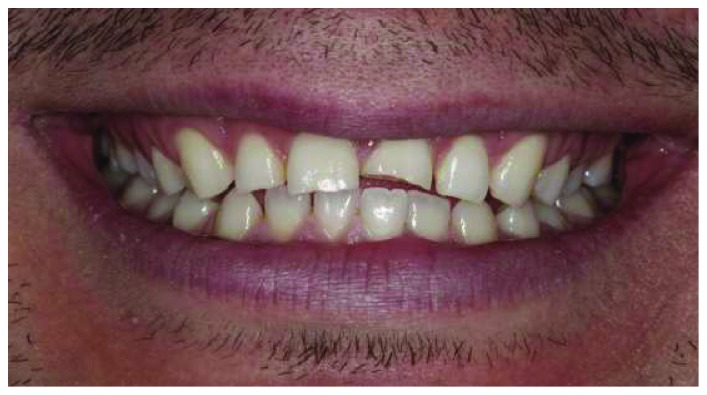
Initial smile of the patient.

**Figure 2:**
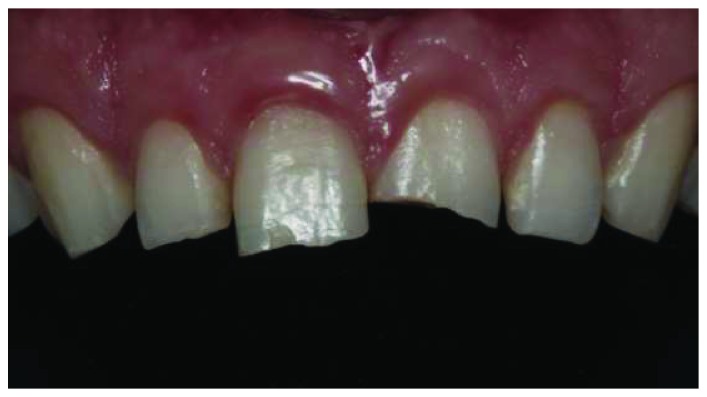
Intraoral view of the fractured anterior teeth.

A healthy 19-year-old male patient attended to our clinic with a chief complaint of maxillary anterior teeth because of the fractures (Figures 1 and 2). All the documents related to the patient including dental anamnesis, intra- and extraoral photographs, and bite registration with impressions from the maxilla and mandible were collected at the first visit. In the dental anamnesis obtained from the patient, it was found that his upper anterior teeth were broken as a result of the fall in childhood. The patient has used his teeth until this age, and he has not had any complaints from his teeth except for the aesthetic appearance. In the intraoral examination, fractured maxillary 12-11-21-22 teeth were found vital and noncarious. In addition, the patient's oral hygiene was good, and the periodontal tissues were healthy. Immediately after the examination in the first visit, the impressions of the maxilla and mandible were taken using alginate. In addition, bite registration was prepared with heated dental wax.

**Figure 3:**
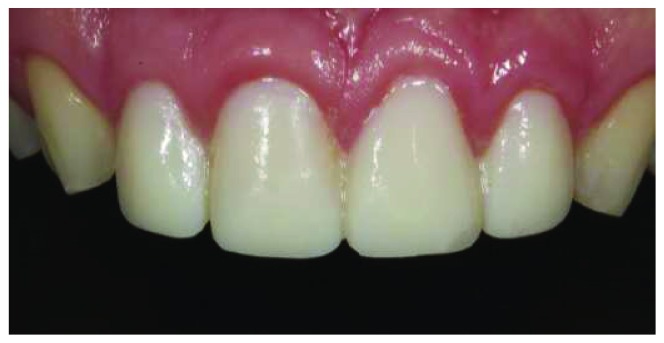
Mock-up made in the mouth through the wax-up prepared in laboratory by free-hand technique.

**Figure 4:**
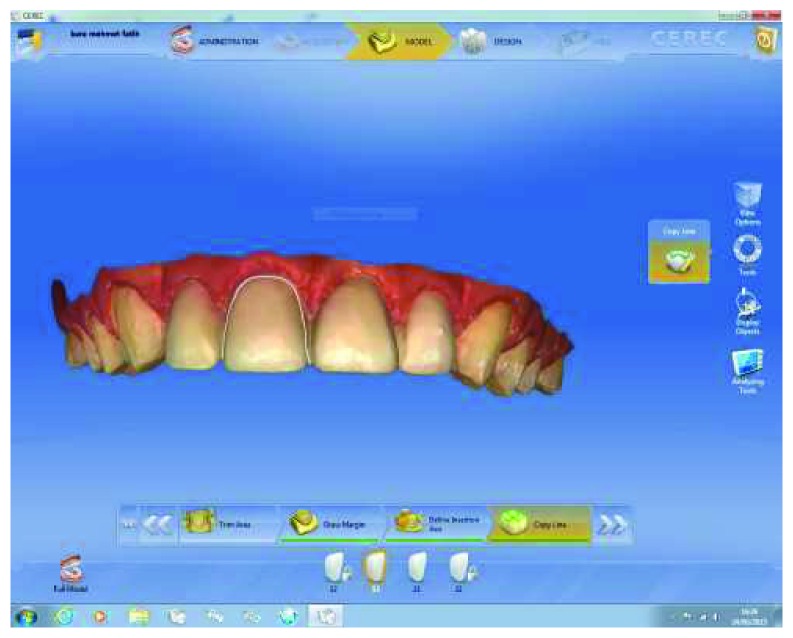
Digital impression via mock-up.

**Figure 5:**
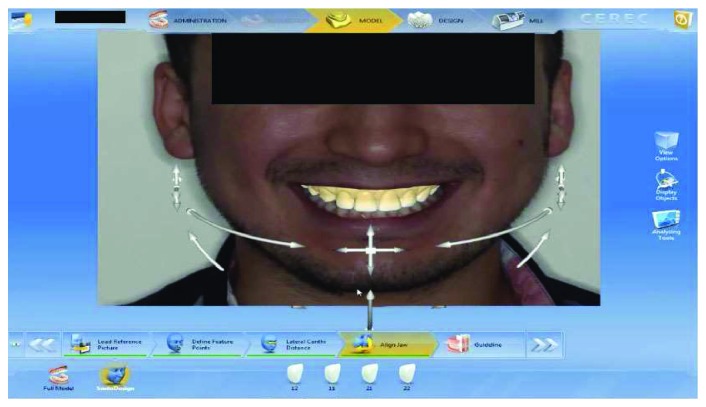
Adaptation of the digital impression with smile design on the patient's photo converted from 2D to 3D by the program.

**Figure 6:**
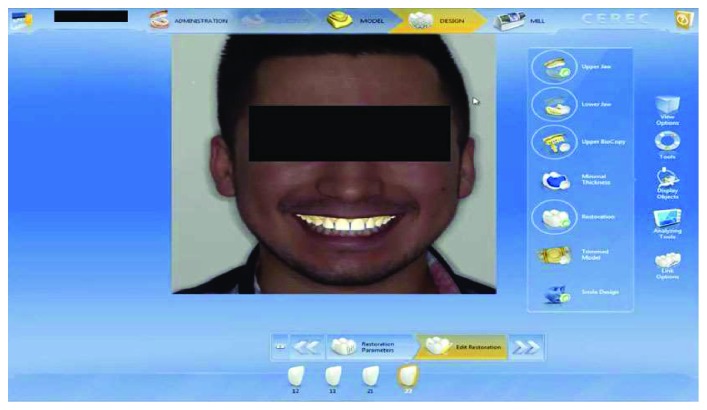
The final appearance of the smile design with the corrected version of the restorations on the digital impression taken with mock-up.

**Figure 7:**
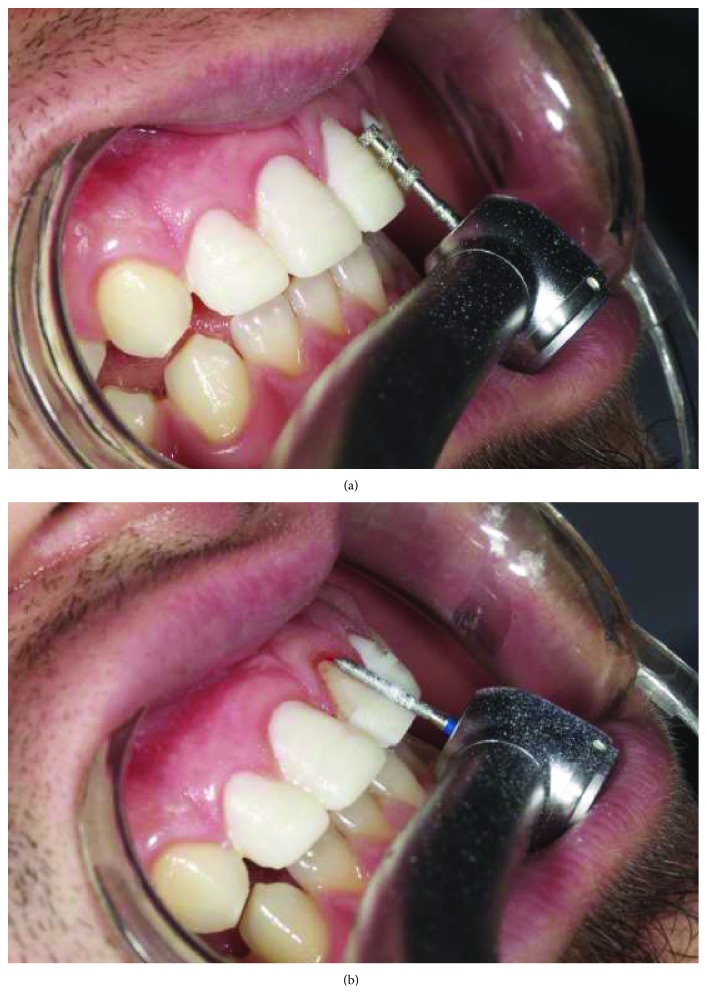
(a, b) Preparation steps on the mock-up after the first digital impression.

**Figure 8:**
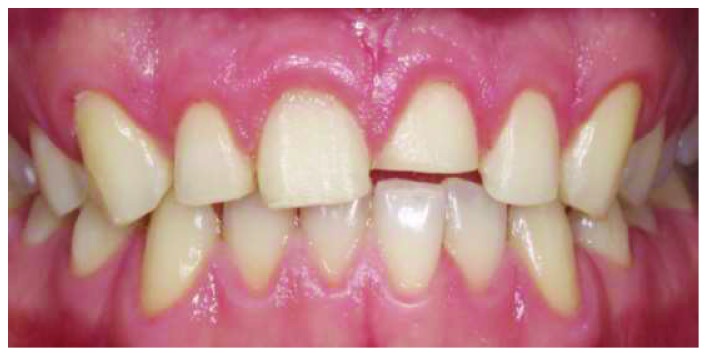
Final view of the almost uncut preparation.

Cast models were provided from the impressions and a wax-up model was prepared by free-hand technique. The wax-up model was duplicated, and vacuum sheet was prepared on the stone cast model for mock-up. At the second appointment, the final volume of the provisional restorations was made with temporary flowable composite resin (Systemp.link, Ivoclar Vivadent) using transparent, rigid, and vacuum-shaped sheets (VacuFormer System, Cavex, Haarlem, Netherlands). The patient was able to preview the estimated finished restoration from the provisional restorations. After patient approval of the mock-up (Figure 3), the first digital impression (Figure 4) was taken on the mock-up from the maxilla with CEREC Omnicam (CAD/CAM, Sirona Dental, Istanbul, Turkey) using the biocopy design mode on the CAD/CAM software. In addition, the patient's photo and digital impression were uploaded to the system, and digital smile design was done on the computer (Figures 5 and 6). The smile design was shown to the patient, and reapproval was obtained (Figure 6). Afterward, preparations were performed over the provisional composite restorations using an operation microscope with a magnification of 40x (Carl Zeiss; Oberkochen, Germany) (Figure 7(a)). Facial surfaces of the teeth were prepared by making depth-orientation grooves (0.3 mm in depth) with a depth preparation diamond bur (Diatech, Coltène Whaledent, Altstätten, Switzerland). The facial reduction was continued with a tapered rounded-end diamond bur (Diatech) until a flat surface was provided under the microscope (Figure 7(b)). All sharp edges and corners were smoothened with an extra-coarse aluminum-oxide polishing disk (OptiDisc, Kerr, Orange, CA, USA) to reduce stress concentrations. Minimal invasive preparations with incisal bevel were provided within the enamel for each tooth. When the teeth were prepared the final shape, the teeth were almost uncut (Figure 8).

**Figure 9:**
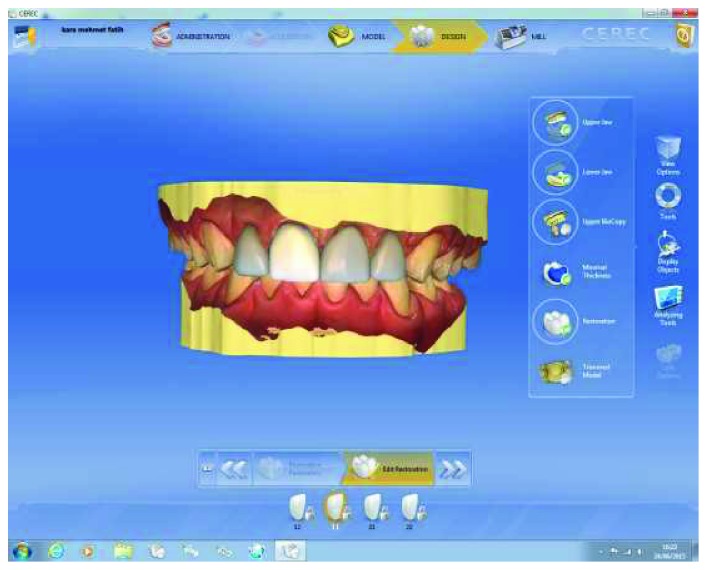
Final view of the restoration design with the corrected restorations on digital impressions completed after the preparation.

**Figure 10:**
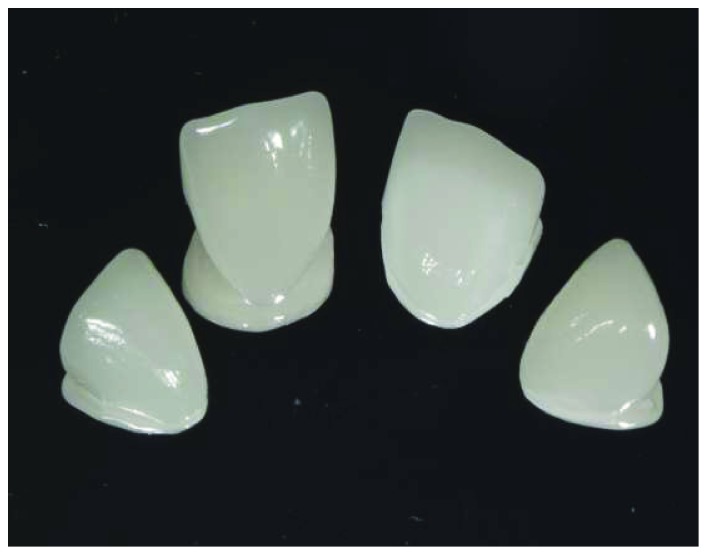
Final view of restorations milled with CAD/CAM.

After finishing the preparations of the teeth, the second digital impression of the maxilla, as well as the first digital impression of the mandible and occlusal bite registration, were taken with the CEREC Omnicam. The mock-up model was copied to the computer, errors on the copy were corrected manually on the computer, and designs of the restorations were completed (Figure 9). The mesiodistal and insicogingival dimensions of the restorations were measured on the computer and were corrected. Symmetry between the teeth was achieved. After completing the restoration design, restorations were milled by CEREC Blocs (Sirona Dental). After the intraoral controls, the restorations were glazed (Figure 10).

A light-curing adhesive resin cement (Variolink Veneer, Ivoclar Vivadent, Schaan, Liechtenstein) was used for the adhesive cementation of the PLVs according to the manufacturers' instructions. The adhesion surfaces of all the veneers were etched with hydrofluoric acid (Vita Ceramics Etch, VITA Zahnfabrik, Bad Säckingen, Germany) for 60 s and subsequently rinsed with water and dried. Monobond S (Ivoclar Vivadent) was applied as a silane for 60 s to the inner surfaces of the veneers. Phosphoric acid (37% Total Etch, Ivoclar Vivadent) was applied to the prepared tooth surfaces including enamel for 30 s and dentine (incisal edge of the left upper central tooth) for 15 s. Adhesive bonding agent (Heliobond, Ivoclar Vivadent) was applied to both the adhesion surfaces of the teeth and the PLVs for 10 s. Resin cement in the selected translucent value (Medium Value 0, Variolink Veneer, Ivoclar Vivadent) was applied to the inner surfaces of the veneers. After these procedures, the PLV restorations were positioned, and excess luting cement was removed with hand instruments and a brush. Before final curing, PLVs were cervically precured for 5 s to remove excess resin cement completely from the cervical and interproximal areas using hand instruments and dental floss without any pressure. For each of the PLVs, these processes were separately made and the PLVs were cemented one by one before the final cure. Final curing was performed according to the manufacturer's instructions for 40 s on each surface (upper- and midbuccal, cervical, mesial, distal, and palatal) with a light-emitting diode polymerizing unit (Elipar S10, 3M ESPE; Neuss, Germany; light output: 1200 mW/cm^2^). Restoration margins were finished and further polished with extrafine diamond finishing burs (Diatech), polishing cups (Kerr HiLuster Plus, Kerr, Orange, CA, USA), and interproximal polishing strips (Sof-Lex Finishing Strips, 3M ESPE, Seefeld, Germany). Finally, the occlusion was checked in protrusive and lateral movements of the mandible.

**Figure 11:**
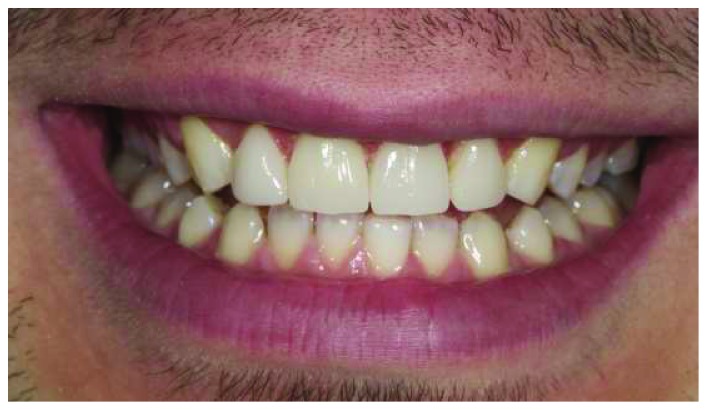
The patient's smile immediately after cementation of the restorations.

**Figure 12:**
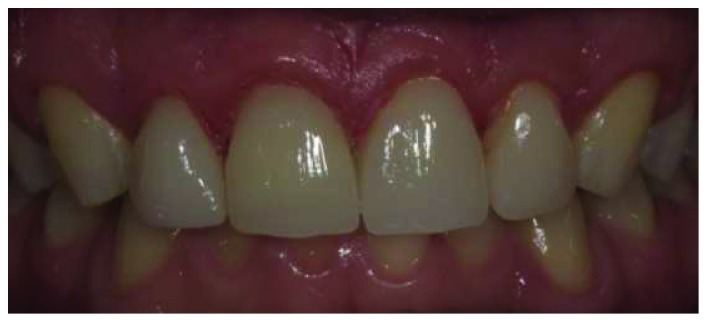
Intraoral appearance of restorations immediately after cementation.

**Figure 13:**
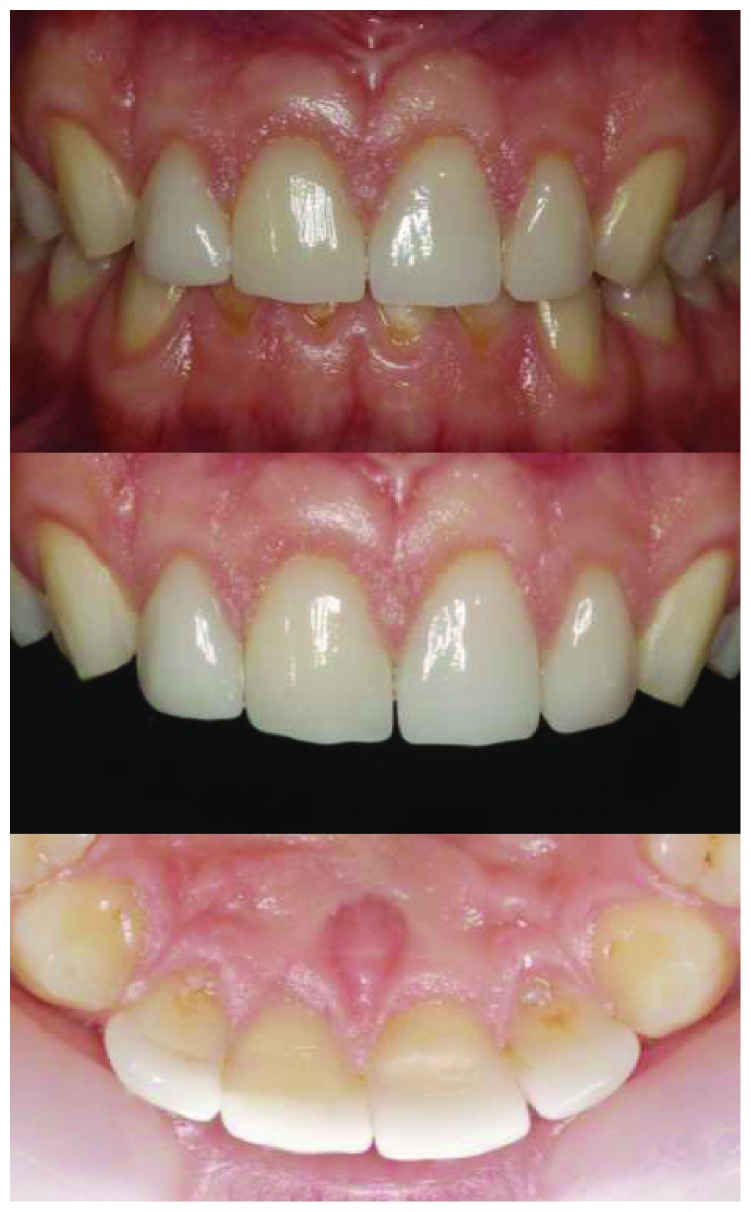
One year follow-up of the restorations.

PLV restorations produced in a single session with CAD/CAM provided the patient's aesthetic rehabilitation and satisfaction quickly (Figures 11 and 12). The patient was recalled after one year, and the restorations were evaluated. PLV restorations were observed to be very good after one year (Figure 13).

## 3. Discussion

This clinical report described how to achieve an esthetic outcome for anterior teeth as minimal invasive as possible with PLVs produced by digital workflow. In addition, to facilitate the computer design, a wax-up model was prepared in the laboratory from the impressions taken immediately in the first visit of the patient. Esthetic rehabilitation of the 4 maxillary anterior teeth was quickly provided with the chairside porcelain laminate veneers produced by CAD/CAM, and the patient satisfaction was provided. Even after one year, the restorations were in very good condition.

The production of a restoration with CAD/CAM includes step by step processes that consist of selection of the tooth, type of restoration, material to be used, selection of the restoration design, taking the digital impressions with intraoral camera, designing the restoration, and finally sending the data to the milling machine to print out the restoration [11]. Three options are available in the software for designing of the restoration: bioreference, biocopy, or biogeneric [12]. In the bioreference project, the design of the restoration incorporates the anatomical features of the corresponding contralateral tooth, if it is present. The biocopy project reproduces the anatomy of the tooth before the preparation, the temporary restoration or a prepared mock-up on the patient, in order to keep as well as to achieve the esthetics and function. In the biogeneric project, the software reads the morphology of the patient's dentition to predict the right form and function. Within the clinical experience of the authors', it can be very difficult to achieve the ideal restoration design with the computer automatically when biogeneric and bioreference are selected, especially in cases where several anterior teeth are needed to be restored with such as PLVs because of inappropriate and/or incomplete anatomic crowns. For this reason, we preferred the biocopy method in this case. In addition, we achieved the perfect restoration design by making corrections on the first digital impression received through the mock-up. Thus, we used mock-up as a step to facilitate the final restoration design.

Different clinical methods can be found in the dental literature to produce porcelain laminate veneers with CAD/CAM. Zandinejad et al. [9] prepared a mock-up in the laboratory before PLVs produced with CAD/CAM and carried out the preparation via this mock-up. After preparation, they sent the digital impressions to the laboratory and produced the PLVs in the laboratory on these impressions. On the other hand, Cattoni et al. [13] used a totally digital process with CAD/CAM from the initial photo shoot to CAD/CAM-milling mock-up. However, the authors did not give any detailed information about the restoration design. Within the knowledge of the authors, there is no published protocol to facilitate computer design in the restoration of anterior unesthetic teeth with PLVs. For this reason, as in this case, time-consuming steps like restoration design can be reduced by the clinical techniques modified according to the patient.

A recent clinical study was published and reported the survival of chairside CAD/CAM porcelain laminate veneers [14]. The authors found that chairside computer-aided design/computer-aided manufacturing porcelain laminate veneers were clinically successful restorations with the mean survival rate of 99.0% after 5 years. Another study evaluating the 2-year clinical success of PLVs produced by the CEREC inLab system in the laboratory reported the success rate of these restorations as 100% and found no significant difference between the porcelain laminate veneers fabricated with CAD/CAM and heat-pressed techniques in terms of marginal and internal adaptation [15]. In both studies, the old software of the CEREC system and BlueCam were used for the restoration design. Therefore, further long-term clinical studies are needed to report the clinical success of chairside PLV restorations produced by new digital techniques. In addition, the introduction of different and/or new clinical techniques into the literature as in this case report would be useful in promoting the applicability of these methods with digital systems.

## 4. Conclusions

PLVs can be produced with CAD/CAM systems in perfect harmony to the teeth with minimal error. The digital workflow facilitates the relationship between dentist and patient. The mock-up model to be prepared can be created and applied by the computer first and can also be implemented by the free-hand technique with heated wax-up before computer design. The clinician can perform whatever technique is more feasible for him or her. A differently modified technique, as here, can help the clinician in designing the restorations with CAD/CAM, especially for the multiple anterior teeth.
